# Standard versus double dose dolutegravir in patients with HIV-associated tuberculosis: a phase 2 non-comparative randomised controlled (RADIANT-TB) trial

**DOI:** 10.12688/wellcomeopenres.16473.1

**Published:** 2021-01-11

**Authors:** Rulan Griesel, Andrew Hill, Graeme Meintjes, Gary Maartens

**Affiliations:** 1 Division of Clinical Pharmacology, Department of Medicine, University of Cape Town, Cape Town, South Africa; 2 Wellcome Centre for Infectious Diseases Research in Africa, Institute of Infectious Diseases and Molecular Medicine, University of Cape Town, Cape Town, South Africa; 3 Department of Pharmacology and Therapeutics, University of Liverpool, Liverpool, UK; 4 Department of Medicine, University of Cape Town, Cape Town, South Africa

**Keywords:** Dolutegravir, rifampicin, drug-drug interaction, tuberculosis

## Abstract

Dolutegravir, a second-generation integrase strand transfer inhibitor (InSTI), is replacing efavirenz as first-line antiretroviral therapy (ART) in low middle-income countries (LMICs). Tuberculosis remains the leading cause of HIV-related morbidity and mortality in LMICs. Rifampicin is a key agent in the treatment of tuberculosis but induces genes involved in dolutegravir metabolism and efflux. The resulting drug-drug interaction (DDI) reduces the exposure of dolutegravir. However, this can be overcome by supplying a supplemental dose of 50 mg dolutegravir 12 hours after the standard daily dose, which is difficult to implement in LMICs.

Four lines of evidence suggest that the supplemental dose may not be necessary: 1) a phase 2 study showed 10 mg of dolutegravir as effective as 50 mg; 2) the prolonged dissociative half-life of dolutegravir after binding to its receptor; 3) a DDI study reported dolutegravir trough concentrations were maintained above its minimum effective concentration when using 50 mg dolutegravir with rifampicin; and 4) virologic outcomes were similar between standard and double dose of raltegravir (a first-generation InSTI) in participants with HIV-associated tuberculosis treated with rifampicin.

We hypothesise that virologic outcomes with standard dose dolutegravir-based ART will be acceptable in patients on rifampicin-based antituberculosis therapy. Here we outline the protocol for a phase 2, non-comparative, randomised, double-blind, placebo-controlled trial of standard versus double dose dolutegravir among adults living with HIV (ART naïve or first-line interrupted) on rifampicin-based antituberculosis therapy. A total of 108 participants will be enrolled from Khayelitsha in Cape Town, South Africa. Follow up will occur over 48 weeks. The primary objective is to assess proportion virological suppression at 24 weeks between groups analysed by modified intention to treat. Participant safety and the emergence of antiretroviral resistance mutations among those with virologic failure will be assessed throughout.

**Trial registratio**ns: ****clinicaltrials.gov NCT03851588 (22/02/2019), SANCTR DOH-27-072020-8159 (03/07/2020).

## List of abbreviations

ABCB1 – ATP binding cassette sub-family B member 1

ABCG2 – ATP binding cassette sub-family G member 2

AIDS – acquired immune deficiency syndrome

ALT – alanine aminotransferase

ART – antiretroviral therapy

AUC – area under the curve

BCRP – breast cancer resistance protein

*β*-hCG – beta-human chorionic gonadotropin

CHC – community health clinic

CI – confidence interval

CRF – case report form

CYP3A4 – Cytochrome P-450 3A4

DAIDS – division of acquired immune deficiency syndrome

DBS – dried blood spot

eGFR – estimated glomerular filtration rate

FDA – United States food and drug administration 

GCP – good clinical practice

HIV – human immunodeficiency virus

HREC – human research ethics committee

IC _90_ – 90% inhibitory concentration

ICF – informed consent form

IDSMC – independent data and safety monitoring committee

InSTI – Integrase strand transfer inhibitors

IRB/IEC – institutional review board/independent ethics committee

mITT – modified intention to treat

IUCD – intrauterine contraceptive device

LDL – lower than detectable limit

LMIC – low middle-income country

MDRD – modification of diet in renal disease

NRTI – nucleoside reverse transcriptase inhibitor

Pgp – P-glycoprotein

PWH – people living with HIV

RCT – randomised controlled trial

RNA – ribonucleic acid

SAE – serious adverse event

SAHPRA – South African health products regulatory authority

SOP – standard operating procedure

TFV-DP – tenofovir-diphosphate

TLD – tenofovir disoproxil fumarate/lamivudine/dolutegravir fixed dose combination

TSC – trial steering committee

UGT1A1 – uridine diphosphate glucuronosyltransferase 1A1

ULN – upper limit of normal

WOCBP – women of childbearing potential

## Introduction

Dolutegravir is being rolled out to replace efavirenz as first-line antiretroviral therapy (ART) in low middle-income countries (LMICs) because it is more effective, better tolerated, and has a considerably higher genetic barrier to resistance ^1^.

Tuberculosis is the commonest cause of human immunodeficiency virus (HIV)-related morbidity and mortality in LMICs, and continues to occur at a higher incidence than in the general population despite normalisation of CD4 counts on ART ^2^. Rifampicin, which is a key component of antituberculosis therapy, induces the following genes that are important in the metabolism and transport of dolutegravir: *UGT1A1*, *ABCB1* (which encodes for P-glycoprotein (Pgp)), *ABCG2* (which encodes for breast cancer resistance protein (BCRP)), and *CYP3A4*. The resulting drug-drug interaction (DDI) between dolutegravir and rifampicin significantly reduces dolutegravir exposure, which can be overcome by increasing the dose of dolutegravir to 50 mg 12 hourly ^3^. Data from the INSPIRING study, a randomised controlled trial (RCT) of double dose dolutegravir versus efavirenz in patients with tuberculosis, reported double dose dolutegravir was well tolerated and rates of virologic suppression were similar in both arms at 24-weeks, but the study wasn’t powered for efficacy comparisons ^4^.

The additional dose of dolutegravir will be difficult to implement in high burden settings where nurses prescribe ART, making complex regimens undesirable, and pharmacies would need to stock dolutegravir as a single tablet as well as the fixed dose combination formulation (tenofovir- lamivudine-dolutegravir (TLD)), increasing the risks of stock outs. Furthermore, the additional dolutegravir tablet increases pill burden and costs. Our experience is that double dose lopinavir-ritonavir, which is recommended with rifampicin-based antituberculosis therapy, is often not implemented in ART programs. For this reason, the South African national guidelines for the roll out of dolutegravir are recommending that efavirenz continue to be used in patients with tuberculosis who are starting ART.

If standard dose dolutegravir is shown to be effective in patients with tuberculosis this would sweep away one of the major barriers to its implementation in LMICs. There are four lines of evidence to support studying standard dose dolutegravir in patients with HIV-associated tuberculosis.

First, there are compelling pharmacokinetic and pharmacodynamic data supporting the therapeutic efficacy of lower dolutegravir exposure. Dolutegravir trough concentrations were found to be the key pharmacokinetic parameter for virologic efficacy in a phase 2a study of different doses of dolutegravir monotherapy ^5^. However, no pharmacokinetic-pharmacodynamic relationships between dolutegravir exposure (including trough concentrations) and virologic outcomes could be established in the phase 2b study (SPRING-1) of different doses of dolutegravir given as part of a combination ART regimen together with dual nucleoside reverse transcriptase inhibitors (NRTIs) ^6^. In SPRING-1 all doses of dolutegravir (10 mg, 25 mg, and 50 mg daily) resulted in similar, high rates of virologic suppression ^6^. The trough concentrations of dolutegravir 50 mg daily were 19 times above the protein-adjusted 90% inhibitory concentration (PA-IC _90_) ^6^, indicating that the drug has a lot of ‘forgiveness’. Therefore, the SPRING-1 study results indicate that lower exposures of dolutegravir have a high likelihood of success.

Second, an important pharmacodynamic characteristic of dolutegravir may mitigate against the emergence of resistance in patients with potentially sub-therapeutic dolutegravir trough concentrations: dolutegravir binds avidly to its receptor, integrase, with a dissociative half-life of 71 hours ^7^.

Third, a DDI study (RADIO) of dolutegravir dosed at 50 mg or 100 mg once daily in healthy volunteers with rifampicin ^8^. Although, as expected, concomitant rifampicin significantly reduced dolutegravir exposure at both doses, all dolutegravir trough concentrations on rifampicin were above the PA-IC _90_ and the median trough concentrations were 2.3 times and 4.3 times above the PA-IC _90_ for the 50 mg and 100 mg dolutegravir doses respectively. The geometric mean trough concentration of the 10 mg dose in SPRING-1 was 0.3 µg/mL; and 0.156 µg/mL and 0.251 µg/mL for the 50 mg and 100 mg dolutegravir doses on rifampicin, respectively in RADIO. The geometric mean ratio of the area under the curve (AUC) of dolutegravir 50 mg once daily with rifampicin versus dolutegravir 50 mg once daily without rifampicin was 44% (90% CI 37, 52), which is similar to the geometric mean ratio of 40% reported for 10 mg versus the 50 mg dolutegravir dose in the SRING-1 study ^6^. As noted above, 10 mg dolutegravir performed as well as 50 mg in SPRING-1 ^6^.

Fourth, exposure to the first-generation InSTI, raltegravir, is also significantly reduced with concomitant rifampicin. A study in healthy volunteers showed that double dose raltegravir overcame the pharmacokinetic DDI with rifampicin ^9^, but a subsequent phase 2 study (ANRS 12 180 Reflate TB) in patients with HIV-associated tuberculosis showed that virologic outcomes were similar with standard and double dose raltegravir ^10^. It is plausible that findings could be similar with dolutegravir.

Our hypothesis is that virologic outcomes with standard dose dolutegravir-based ART will be acceptable in patients on rifampicin-based antituberculosis therapy. If the proportion of participants who achieve virological suppression on standard dose dolutegravir is acceptable, this would pave the way for a phase 3 trial of dolutegravir 50 mg daily versus an appropriate standard of care regimen, like efavirenz-based ART, in patients with HIV-associated tuberculosis. A variety of safety measures will be put in place to ensure that no harm will come to participants.

## Protocol

### Objectives

The primary objective of the **R**ifampicin **A**nd **D**olutegravir **I**nvestig **A**tion of **N**ovel **T**reatment dosing in **T**u **B**erculosis (RADIANT-TB) trial is to assess the proportion of people living with HIV (PLWH) on rifampicin-based antituberculosis therapy with virologic suppression between standard versus double-dose dolutegravir-based ART regimen at 24 weeks.

Secondary objectives among PLWH on rifampicin-based antituberculosis therapy in the trial include: 1) the proportion of virologic suppression at differing time points after initiating a standard versus double-dose dolutegravir-based regimen; 2) assess the effect of ART first-line interruption (versus ART-naïve) on virologic outcomes among participants initiating standard versus double-dose dolutegravir-based regimen; 3) assess the difference in immune reconstitution at 24 weeks between standard versus double-dose dolutegravir-based regimen; 4) determine the effect of rifampicin-based antituberculosis therapy on dolutegravir trough concentrations between a standard versus double-dose dolutegravir-based regimen; 5) assess the adherence of participants in the trial; 6) assess clinical safety and tolerability of a standard versus double-dose dolutegravir-based regimen; 7) determine the emergence of antiretroviral resistance mutations among participants with virologic failure between standard versus double-dose dolutegravir-based regimen. 

Table 1 reports the trial objectives and related outcome measures in more detail.

**Table 1.** Study objectives and outcomes.

Objectives	Outcome Measures	Timepoint(s) of evaluation of this outcome measure
**Primary** The proportion of PLWH on rifampicin-based anti-tuberculosis therapy with virologic suppression between standard versus double-dose dolutegravir-based regimen.	Proportion with HIV viral load <50 copies/mL at 24 weeks analysed by mITT, which includes all participants who received at least one dose of dolutegravir, and according to the FDA snapshot algorithm.	24 weeks
**Secondary** 1) The proportion of virologic suppression at varying time points after initiating a standard versus double-dose dolutegravir-based regimen among PLWH on rifampicin-based antituberculosis.	Proportion with HIV viral load <50 copies/mL at 12 weeks analysed mITT Proportion with HIV viral load <50 copies/mL at 48 weeks analysed mITT Proportion with HIV viral load <50 copies/mL at 12, 24 and 48 weeks analysed per protocol	12, 24, 48 weeks
2) Assess the effect of ART interruption (versus ART-naïve) on virologic outcomes among PLWH on rifampicin-based antituberculosis therapy initiating standard versus double- dose dolutegravir-based regimen.	All primary and secondary virological endpoints stratified by baseline ART-naïve or first-line ART interruption status	12, 24, 48 weeks
3) Assess the difference in immune reconstitution at 24 weeks between standard versus double-dose dolutegravir-based regimen among PLWH on rifampicin-based antituberculosis.	Change in CD4 count from screening at week 24	24 weeks
4) Determine the effect of rifampicin-based antituberculosis therapy on dolutegravir trough concentrations between a standard versus double-dose dolutegravir-based regimen among PLWH initiating ART	Proportion with dolutegravir trough concentrations above the PA-IC _90_ at weeks 4, 8, 12, 24, and 48.	4, 8, 12, 24, and 48 weeks
5) Assess adherence of participants in the trial.	TFV-DP DBSs, which is an objective medium-term ART adherence measure, at weeks 4, 8, 12 ,24 and 48.	4, 8, 12 ,24 and 48 weeks
6) Assess clinical safety and tolerability of a standard versus double-dose dolutegravir-based regimen among PLWH on rifampicin-based antituberculosis therapy initiating ART	Grade 3 or 4 drug-related adverse events Change from baseline of sleep assessment and mental health questionnaires Serious adverse events Adverse events requiring discontinuation of any drug in the ART regimen	Continuously
7) Determine the emergence of antiretroviral resistance mutations among participants with virologic failure between standard versus double-dose dolutegravir-based regimen among PLWH on rifampicin-based antituberculosis therapy initiating ART.	Emergence of antiretroviral resistance mutations in participants with virologic failure	Continuously

HIV: human immunodeficiency virus; PLWH: people living with HIV; mITT: modified intention to treat; FDA: United States food and drug administration; ART: antiretroviral therapy; PA-IC _90_: protein adjusted 90% inhibitory concentration; TFV-DP DBS: tenofovir diphosphate dried blood spot

### Study design

An exploratory phase 2b randomised (1:1), double-blind, placebo-controlled, parallel group trial of TLD daily with an additional 50 mg dose of dolutegravir or matching placebo taken 12 hours later among PLWH on rifampicin-based antituberculosis therapy.

### Study sites

The study will be conducted at a primary care clinic in Khayelitsha, Cape Town, South Africa (Khayelitsha Site B/Ubuntu CHC) – primary research facility. Eligible patients will also be referred from 2 satellite sites (Michael Mapongwana CHC, Cape Town, South Africa and Nolungile/Site C CHC, Cape Town, South Africa) for screening and enrolment at the primary research facility.

### Trial participants

The trial will aim to enroll 108 participants (see sample size calculation). Patients with HIV-associated tuberculosis will be approached to obtain written informed consent for screening. If eligible (see inclusion and exclusion criteria) they will be invited to participate in the study and provided with the informed consent document.

### Recruitment strategies

Clinicians at the primary care clinic and satellite referral centres will be approached and informed of the trial. They will be requested to refer appropriate patients to our trial for screening. Posters of trial eligibility criteria will be disseminated among clinicians and cellular application groups will be used to send reminders. The trial will give regular feedback sessions to update clinicians on the progress and encourage continued referral.

### Inclusion and exclusion criteria

To be included in the trial a participant must: 1) have given written informed consent to participate; 2) be at least 18 years old; 3) have HIV-1 infection with a documented screening plasma HIV-1 ribonucleic acid (RNA) >1000 copies/mL; 4) be ART-naïve or have interrupted first-line ART (criteria for interruption: on ART <6 months prior to interruption or virologically suppressed [<50 copies/mL or LDL] <6 months prior to interruption); 5) must be on rifampicin-based antituberculosis therapy for less than 3 months; 6) a screening CD4 count greater than >100 cells/µL; and 7) women of childbearing potential (WOCBP) should be willing to use appropriate contraception. Full exclusion criteria are outlined in Table 2.

**Table 2.** Eligibility criteria.

Inclusion criteria:
• ≥18 years old
• HIV-1 infection as documented by screening plasma HIV-1 RNA >1000 copies/mL;
• ART-naïve (short-term antiretroviral use for prevention of mother-to-child transmission will be allowed) **OR** • First-line ART treatment interrupters on ART <6 months prior to interruption or virologically suppressed (<50 copies/mL or LDL) <6 months prior to interruption
• On rifampicin-based therapy for tuberculosis for <3 months
• CD4 counts >100 cells/µL
• WOCBP willing to use adequate contraception (defined as either an IUCD or hormonal contraception as per national guidelines)
Exclusion criteria:
• Pregnant/breastfeeding
• eGFR <60 mL/min/1.73 m ^2^ (calculated by the MDRD study)
• ALT >3 times ULN
• Allergy or intolerance to one of the drugs in regimen
• Concomitant medication known to significantly reduce or increase dolutegravir exposure (except rifampicin)
• Active psychiatric disease or substance abuse
• On treatment for active AIDS-defining condition other than tuberculosis (participants on maintenance therapy may be enrolled)
• Malignancy
• Any other clinical condition that in the opinion of an investigator puts the patient at increased risk of participating in the study.

HIV: human immunodeficiency virus; RNA: ribonucleic acid; ART: antiretroviral therapy; LDL: lower than detectable limit; WOCBP: woman of child-bearing potential; IUCD: intrauterine contraceptive device; eGFR: estimated glomerular filtration, MDRD: modification of diet in renal disease; ALT alanine aminotransferase; ULN: upper limit of normal; AIDS: acquired immune deficiency syndrome.

First-line ART interrupters will be enrolled into the study to make the findings more generalisable – in our experience at the research facility the majority of PLWH with associated tuberculosis not on ART have interrupted therapy rather than being ART-naïve. There is a potential risk of impaired virologic response in participants who have interrupted treatment as they may harbour NRTI resistance mutations; however, this risk will be mitigated by only selecting participants who have a low risk of NRTI viral resistance mutations (either on ART for <6 months at the time of interruption or a suppressed viral load <6 months before the time of interruption).

### Informed consent

A study specific informed consent form (ICF) ^11^ will include all elements required by good clinical practice (GCP) as well as all local ethics and regulatory requirements. The principal investigator’s trained designees (clinical research workers/counselors or research nurses) will ensure that participants are fully informed about the purpose, potential risks, and other critical issues regarding the clinical trial in which they will participate and that their participation is voluntary. The informed consent process will be conducted as per the trial’s standard operating procedures (SOPs). The informed consent process will occur in a private location in a language of the patient’s choice. Only patients with full decisional capacity will have the informed consent process performed and understanding of the ICF and trial procedures will be assessed before signing. A copy of the signed ICF will be given to the participant. If the patient is illiterate, an impartial witness must be present during the entire consent process. In such cases a thumb print may be used as a signature. The ICF will be updated with any pertinent information that becomes available during the study. Currently no other analyses of stored blood samples are planned, however participants are expected to give clear written consent to have samples stored should they agree (separate signature section on ICF).

### Randomisation and blinding

Participants will be assigned either placebo or the supplemental dose of dolutegravir in a 1:1 allocation using permuted block randomisation. Two separate randomization schedules will be generated by an independent pharmacist utilizing a computerised random number generator (the Mersenne Twister algorithm (MT19937) in Excel 2016). Participants will be stratified to either one of the two schedules based on their baseline ART-naïve or first-line treatment interruption status. 

The independent pharmacist will share the randomisation schedules with the study pharmacist who will prepare individually sealed opaque envelopes that contain the randomisation treatment number and assigned treatment. The sealed envelopes will be kept safe and secure within a locked cupboard in the study pharmacy to which only the study site pharmacist and authorised designees will have access. Allocation concealment will be maintained as none of the investigators, other study team members, or participants will have access to the randomisation schedules or sealed envelopes.

All patients who give consent for participation and fulfil the eligibility criteria will be enrolled. A prescription for TLD and trial medication (supplemental dolutegravir or placebo) will be filled and sent to the study site pharmacist. The study site pharmacist will verify enrolment and randomise the participant by drawing the next available sequentially numbered sealed envelope from the appropriate randomisation schedule. The participant will be assigned the relevant randomisation treatment number, the sealed envelope opened, and the assigned trial medication allocated. A second trial pharmacist will verify randomisation and dispensing of TLD and trial medication.

The investigators, other study team members, and participants will remain blinded to the treatment allocation. Blinding will be ensured by the use of a placebo identical to the active drug (dolutegravir 50 mg, Myltega®; Mylan, Canonsburg, Pennsylvania, USA). Only the study site pharmacist and trial statistician will be unblinded; none of whom will be allowed to share the randomisation schedule with investigators, other study team members, or participants. Emergency unblinding of the treatment allocation should only occur under exceptional circumstances when this information is deemed essential for ongoing clinical management by an attending clinician. The principal investigator will be responsible for the final decision on emergency unblinding and this will be made in consultation with the trial steering committee (TSC). A detailed SOP for emergency unblinding has been developed: only the principal investigator will be allowed to contact the study site pharmacist to obtain a specific treatment allocation. All events of emergency unblinding will be recorded and reported to the institutional review board/independent ethics committee (IRB/IEC), regulatory authority, and independent drug safety monitoring committee (IDSMC).

### Trial procedures

The study procedures are detailed in Table 3. Participants will be involved in the trial for 48 weeks, excluding a screening period, which should not be longer than 8 weeks.

**Table 3.** Schedule of events.

	Visit 1 Screening	Visit 2 Baseline	Visit 3	Visit 4	Visit 5	Visit 6	Visit 7	Visit 8	Visit 9	Visit 10
**Week**	-8 weeks	0	4	8	12	16	20	24	48	52
**Time window**			+/- 16 days	+/- 16 days	+/- 16 days	+/- 16 days	+/- 16 days	+/- 16 days	+/- 16 days	
**Informed consent**	X									
**Review inclusions & exclusion** **criteria**	X	X								
**Medical history**	X									
**Vital signs**	X	X	X	X	X	X	X	X	X	
**Physical exam**	X	X	X	X	X	X	X	X	X	
**Randomisation**		X								
**Placebo or dolutegravir** **administration**		Continues till 2 weeks after antituberculosis medication is finished.								
**Adverse events**		X	X	X	X	X	X	X	X	
**Mental health questionnaire**		X			X			X	X	
**Sleep assessment ** **questionnaire**		X	X	X	X	X	X	X	X	
**Pregnancy test (urine β-HCG)**	X	X	X	X	X	X	X	X	X	
**Contraception for WOCBP**	X	X	X	X	X	X	X	X	X	
**Potassium creatinine,** **bilirubin, & ALT**	X		X	X	X			X	X	
**HIV-1 RNA**	X		X	X	X			X	X	
**CD4 count**	X							X	X	
**Dolutegravir trough**			X	X	X			X	X	
**TFV-DP DBS**			X	X	X			X	X	
**Plasma for storage**		X	X	X	X			X	X	
**Antiretroviral resistance** **testing**		If viral load is >1000 copies/mL at week 24 or if viral load was suppressed and then rebounded to >1000, resistance testing will be performed on a stored plasma sample, and compared to a baseline stored sample								
**Referral to public sector care**										X

β-HCG: beta human chorionic gonadotropin; WOCBP: women of child-bearing potential; ALT: alanine aminotransferase; RNA: ribonucleic acid; TFV-DP DBS: tenofovir diphosphate dried blood spot.

***Screening visit.*** Eligible patients will need to start ART within 2–12 weeks after starting antituberculosis therapy. All eligible patients will sign informed consent. Medical history, method of tuberculosis diagnosis, vitals (all instrumentation used to perform weight and vital signs will be kept in a functional state and have regular calibration), physical exam, and safety blood monitoring. WOCBP should have a urine pregnancy test and referred for appropriate contraception. Urine pregnancy testing will be performed using the Clinitek hCG® pregnancy test analyser (Siemens Healthcare Diagnostics, Tarrytown, NY): a chromatographic immunoassay for the rapid determination of beta-human chorionic gonadotropin (β-hCG) in urine. If a test is positive or indeterminant, a serum β-hCG will be performed.

***Baseline visit.*** Participants who are eligible for the study will return for a baseline visit (within 8 weeks of screening). A mental health assessment questionnaire ^12^: the validated modified MINI screen (MMS) ^13^ will be conducted on each enrolled participant. Furthermore, a basic sleep assessment followed by the validated insomnia severity index (ISI) questionnaire ^14^ will be conducted ^15^. All questionnaires will be performed by adequately trained counsellors in a language that the participant understands. All eligibility criteria will be checked, and at the investigator’s discretion, the participant will be enrolled into the study. A blood sample for storage will be taken if the participant consented to stored samples. The participant will be randomised, and treatment dispensed by the study site pharmacist.

***Follow up visits.*** Participants will return every 4 weeks for a physical exam, adverse event check (unsolicited), sleep assessment, mental health questionnaire (every 12 weeks) and serum biochemical safety tests (alanine aminotransferase (ALT), bilirubin, creatinine, and potassium). HIV-1 RNA (viral load) will be done at weeks 4, 8, 12, 24 and 48. An additional sample for storage will be taken with each viral load sample, this will be for antiretroviral resistance testing should it become necessary. Contraception use will be checked at every visit for WOCBP. Dolutegravir trough concentrations and tenofovir-diphosphate (TFV-DP) dried blood spot (DBS) testing will be done at weeks 4, 8, 12, 24 and 48.

***End of study visit.*** After 48 weeks, the participant will return to the site for a final study visit. They will be referred out to their local clinic and dispensed with one month of TLD. A follow up call to the participant will be made to ensure that they have accessed continuing care.

***Adherence assessment.*** Adherence will be addressed at enrolment and each follow up visit. Participants will complete a patient adherence plan and counseling will follow the adherence guidelines for HIV, tuberculosis, and non-communicable diseases as outlined by the South African National Department of Health. Important aspects of adherence that will be covered include: dose timing, medication storage, taking medication with a meal, what to do in the event of a missed dose, discussion on potential side-effects, and reminder methods.

Adherence will be assessed by pill count at each follow up visit. However, this is not the most reliable method to assess adherence, and for this reason dolutegravir trough concentrations and TFV-DP DBS will be determined at regular intervals as outlined in Table 3. The Clinical Pharmacology Laboratory at University of Cape Town, Cape Town, South Africa has developed validated assays for both of these analytes on liquid chromatography-mass spectrometry ^16^. We will use thresholds of TDF-DP concentrations in DBS that have been determined to predict different adherence levels ^17^.

***Participant retention.*** Once a participant is enrolled and randomised, the study site will make every reasonable attempt to follow the participant for the duration of the study period, this will include systematic methods of reminders and contacting participants and limiting participant burden related to follow up visits and procedures. Participants will receive reimbursement for travel costs every time they attend a visit at the study site. It is projected that the rate of loss to follow up will be at most 10%. Study site staff are responsible for developing and implementing SOPs to achieve this level of follow up.

***Withdrawal and loss to follow up.*** Participants reserve the right to withdraw from the study at any stage, without an obligation to provide reasons for their withdrawal. Participants may choose to stop treatment but remain in study follow up. Participants may also withdraw their consent, meaning that they wish to withdraw from the study completely. Should a participant voluntarily withdraw their consent after receiving study treatment, the study team will attempt to ascertain the reason for withdrawal and encourage the patient to complete a withdrawal/final study visit as soon as possible, while respecting the participants decision. All relevant discussions with the participant will be documented. Data collected up to the time of stopping treatment/consent withdrawal will be used in the analysis. No further data or samples would be collected after consent withdrawal.

In addition, the investigator may discontinue a participant from the trial treatment at any time for any reason including, but not limited to: pregnancy; ineligibility (either arising during the trial or retrospectively having been overlooked at screening); significant protocol deviation or significant non-compliance with treatment or trial requirements; an adverse event which requires discontinuation of the trial medication or results in inability to continue to comply with trial procedures; and disease progression which requires discontinuation of the trial medication or results in inability to continue to comply with trial procedures. In such case a withdrawal/final study visit should be performed whenever possible and the type of withdrawal and reason for withdrawal recorded.

Participants who develop adverse events deemed related to dolutegravir that requires discontinuation will be withdrawn from the study. Participant safety will be ensured by continued follow up after withdrawal from the study. Their data will be part of the primary and secondary outcomes in the intention-to-treat analysis. Switches of one or both of the dual NRTIs for toxicity will be allowed.

Participants who become pregnant on the study will be withdrawn and the dolutegravir switched to efavirenz. Pregnancy outcomes for the participant and the infant will be recorded. This is especially important in this study as a recent report has implicated dolutegravir as a cause of neural tube defects ^18^.

All participants who withdraw from the study will be referred to their local HIV clinic for ongoing care.

Participants who miss two consecutive visits will be deemed lost to follow up and withdrawn from the study. All possible efforts will be made to ensure participants attend the final safety assessment. These include contacting participants telephonically and rescheduling a visit if feasible. All relevant discussions with the participant will be documented. Data collected up to the time of lost to follow up will be used in the analysis.

### Interventions

***Trial medication.*** Dolutegravir 50 mg tablet (Myltega®; Mylan, Canonsburg, Pennsylvania, USA) or matching placebo (Mylan, Canonsburg, Pennsylvania, USA) will be administered orally 12 hours after the morning fixed dose combination of TLD (tenofovir disoproxil fumarate 300 mg, lamivudine 300 mg, dolutegravir 50 mg) from the start of the study until 2 weeks after completing rifampicin-based antituberculosis therapy. The study pharmacist will prepare, package, and label dolutegravir/placebo for each participant according to the randomisation code and dispense a month’s supply to be taken together with the fixed dose combination. The ingredients of the placebo will be the standard excipients found in dolutegravir 50 mg tablet without the active ingredient. None of these will have any biological effect. TLD will be obtained from the South African National Department of Health stock but dispensed by the study pharmacy.

***Concomitant treatments.*** Rifampicin-based antituberculosis therapy, and co-trimoxazole preventative therapy will be supplied by the community health clinics as part of standard of care.

Medication that needs to be changed/used with caution during enrolment include:

1. Polyvalent cations (Mg, Fe, Ca, Al, Zn) e.g. antacids, sucralfate, supplements – preferably avoid, but if taken, dolutegravir should be taken either 2 hours before or 6 hours after.
2. Anticonvulsants: carbamazepine, phenobarbital, phenytoin – no co-administration. Lamotrigine, levetiracetam, and topiramate can be used.
3. Metformin: maximum dose is 500 mg 12 hourly when taken with dolutegravir.

Dolutegravir has recently been implicated as a potential cause of neural tube defects ^18^. As per the exclusion criteria above, WOCBP must be on adequate, reliable contraception, defined as an intrauterine contraceptive device (IUCD) or hormonal contraception as per national guidelines. WOCBP in the study who do not have an IUCD will be given hormonal contraception for the duration of the study. As rifampicin is a known potent inducer, thereby reducing the effectiveness of oral and implantable contraceptives by enhancing their metabolism, we recommend the use of injectable progesterone-only contraceptives such as medroxyprogesterone acetate or norethisterone enantate.

### Assessment of safety

***Adverse events.*** An adverse event is any untoward medical occurrence developing in participants from the first dose of study drug until the end of the study or developing after this period and thought to be not related, possibly, probably, or likely related to the study drug.

Drug-related adverse events reported by participants or observed by the investigator, and any remedial actions, will be recorded in the source documents and the participant’s case report form (CRF). The nature of the event, the time of onset, duration, severity (Division of AIDS table for grading the severity of adult and pediatric adverse events) ^19^ and causality assessment will be recorded in the individual CRFs and included in the study report.

Any serious adverse event (SAE) (defined as resulting in death, being life threatening, requiring hospitalization, resulting in significant or persistent disability/incapacity, or jeopardizing the participant, or requiring intervention to prevent significant or persistent damage or disability) will be reported to the principal investigator immediately. SAEs and all suspected unexpected serious adverse reactions (SUSARs) to be related to the study drugs, will be reported to the IRB/IEC and regulatory authority according to their current guidelines and in line with GCP guidelines.

***Collection and documentation of adverse events.*** The occurrence of adverse events will be solicited from participants at each study visit using a standardized adverse event collection tool with which participants will be encouraged to report any untoward change in their condition and questioned about specific adverse events related to insomnia (ISI questionnaire), new or worsening neuropsychiatric symptoms (MMS questionnaire), and symptoms of hepatotoxicity. Participants will also be asked to contact the study team immediately should they develop any untoward changes in their medical condition.

***Renal function impairment.*** If the estimated glomerular filtration rate (eGFR) declines to <50 mL/min then tenofovir disoproxil fumarate will be discontinued and replaced with abacavir as per South African national guidelines. Tenofovir disoproxil fumarate may be reintroduced if there is an alternative explanation for the decline in renal function once the eGFR has increased to >60 mL/min.

***Suspected drug-induced liver injury.*** Dolutegravir, cotrimoxazole, and several antituberculosis drugs (isoniazid, rifampicin, and pyrazinamide) can cause drug-induced liver injury.

Liver chemistry threshold stopping criteria have been designed to assure participant safety and to evaluate liver event aetiology during administration of dolutegravir and the follow up period. Dolutegravir, cotrimoxazole, and antituberculosis therapy (if the participant is still taking this) will be stopped if any of the following liver chemistry criteria are met:

- ALT ≥3 times upper limit of normal (ULN) (or 3 times baseline ALT if this was above ULN) and bilirubin ≥2 times ULN
- ALT ≥3 times ULN (or 3 times baseline ALT if this was above ULN) with symptoms of acute hepatitis or hypersensitivity such as fatigue, nausea, vomiting, right upper quadrant pain or tenderness, fever, or rash;
- ALT ≥5 times ULN irrespective of bilirubin and symptoms

Participants who develop ALT ≥5 times ULN should be followed weekly until resolution or stabilization (ALT <5 times ULN on 2 consecutive evaluations). Participants should not restart dolutegravir due to the risk of a recurrent reaction unless an alternative diagnosis is made for the liver chemistry abnormalities (e.g. viral hepatitis). 

***Viral load monitoring and detection of antiretroviral resistance.*** Frequent viral load monitoring will be done during the study as per the schedule of events ( Table 3). Antiretroviral resistance testing will be done in participants who do not have virologic suppression at week 24 or in participants who suppress their viral loads and then subsequently rebound. Resistance testing will also be done on stored plasma from the baseline visit in participants who have antiretroviral resistance mutations to distinguish emergent from pre-treatment resistance. Participants who have antiretroviral resistance mutations will be assigned an appropriate ART regimen, using antiretrovirals available in national guidelines (which includes efavirenz, zidovudine, and lopinavir-ritonavir).

### Statistical considerations and analyses

***Power and sample size.*** We assume that 85% of participants will achieve virologic suppression at week 24. With 49 patients per group the lower 95% CI of virological suppression at week 24 would exceed 70% (actual value is 73%) with power of 80% and α of 5% (one-sided test). We selected this lower 95% CI of virologic suppression based on the outcomes in two randomised controlled trials with efavirenz-based ART in patients with HIV-associated tuberculosis, which achieved suppression of 74% and 70% at 48 weeks ^20^, 21. Assuming a 10% rate of loss to follow we plan to enroll 54 participants per arm. The study is not powered for formal comparison of efficacy between the two arms.

***Primary endpoint.*** Proportion with HIV viral load <50 copies/mL at 24 weeks analysed by modified intention to treat (mITT), which includes all participants who received at least one dose of dolutegravir, and according to the Food and Drug Association (FDA) of USA snapshot algorithm. The FDA snapshot algorithm regards the following categories of participants as failures: HIV viral load ≥50 copies/ml, missing HIV viral load within the visit window, or those who have discontinued ART. Switching of the NRTI component of the ART regimen for intolerance or adverse event will not be regarded as failure but switching or stopping of dolutegravir will be regarded as failure. Switching due to pregnancy will not be regarded as failure.

***Secondary endpoints***

- Proportion with HIV viral load <50 copies/mL at 12 weeks analysed mITT
- Proportion with HIV viral load <50 copies/mL at 48 weeks analysed mITT
- Proportion with HIV viral load <50 copies/mL at 12, 24 and 48 weeks analysed per protocol
- Stratification of primary and secondary virologic outcomes by baseline ART-naïve versus first-line treatment interruption status
- Change in CD4 count from screening at week 24
- Proportion with dolutegravir trough concentrations above the PA-IC _90_ at weeks 4, 8, 12, 24, and 48.
- TFV-DP DBS – which is an objective medium-term ART adherence measure, at weeks 4, 8, 12, 24 and 48.
- Grade 3 or 4 drug-related adverse events
- Change from baseline of sleep assessment and mental health questionnaires
- SAEs
- Adverse events requiring discontinuation of any drug in the ART regimen
- Emergence of antiretroviral resistance mutations in participants with virologic failure (resistance testing will be done on stored plasma at baseline in those with on study antiretroviral resistance mutations to distinguish emergent from pre-treatment resistance)

***Analysis plan.*** Statistical analyses will be performed using Stata version 16.0 (Stata Corp, College Station, Texas, USA). The proportions of participants with virologic suppression will be determined with 95% CIs. mITT and per protocol analyses will be performed as outlined in the primary and secondary endpoints above. Between-group differences for secondary endpoints will be analysed with chi-squared tests (or Fisher’s exact tests if the number in any cell is ≤5) for categorical data and with Wilcoxon rank sum tests for continuous data. As noted above in the sample size section, no formal between group comparisons will be made of the primary endpoint as we do not have sufficient power. Data from participants who do not complete the study will be used for secondary endpoint analyses.

### Data management

Upon screening participants will receive a screening number and folder. The screening document will identify the patient based on his/her hospital folder number only. At the enrolment visit participants will receive a participant identification number and participant study folder, replacing the screening number and folder. The participant identification number will completely de-identify the participant. Participant study folders are to be stored in numerical order in a secure and accessible place. Participant study folders will be retained in storage for a period of 3 years after completion of the trial.

Study visit source documents and CRFs will be completed during the study visit. Data will be captured from CRFs on an online electronic data capturing system and stored on University of Cape Town servers. The electronic data capturing system will use a password protected platform and grant users access rights based on trial status. The system can be used by multiple users simultaneously and all users’ actions are traceable throughout the trial. The principal investigator has overall responsibility for ensuring the data collected are complete, accurate, and recorded in a timely manner. Confidentiality of records must be protected, respecting the privacy and confidentiality rules in accordance with the applicable regulatory and GCP requirements.

Only the unblinded trial statistician will have full access to the data set during the trial. Once the trial is closed to enrolment and follow up completed the principal investigator will have access to the cleaned unblinded data set. 

### Samples

***Sample handling.*** The samples to be collected are listed in Table 3. Details of sample collection, processing, transport, and storage are available in the trial Sample Management SOP.

The following samples will be analysed/stored at the following laboratories:

- Urine pregnancy test (urine β-hCG) – on site analysis as previously mentioned
- Potassium, creatinine, bilirubin, and ALT; VL; and CD4 count – South African National Health Laboratory Services (NHLS) at Groote Schuur Academic Hospital in Cape Town, South Africa (SA National Accreditation System - ISO 15189:2007)
- Dolutegravir trough and TFV-DP DBS concentrations – will initially be stored at -80°C at the Wellcome Centre for Infectious Diseases Research in Africa laboratory, Faculty of Health Sciences, University of Cape Town, Cape Town, South Africa. At the end of the trial these samples will be retrospectively analysed by the Clinical Pharmacology Laboratory, Faculty of Health Sciences, University of Cape Town, Cape Town, South Africa (SA National Accreditation System - ISO/IEC 17025:2005). The laboratory has participated in the Clinical Pharmacology Quality Assurance (CPQA) external quality control program under a contract with the Division of AIDS of the National Institute of Allergy and Infectious Diseases. The assays were CPQA approved.
- Plasma for storage – plasma will be stored for potential future analysis or antiretroviral resistance testing should the need occur. Samples will be stored at -80°C at the Wellcome Centre for Infectious Diseases Research in Africa laboratory, Faculty of Health Sciences, University of Cape Town, Cape Town, South Africa.
- Antiretroviral resistance testing – should the need occur analyses will be done by the South African National Health Laboratory Services (NHLS), Division of Virology at Tygerberg Hospital, Cape Town, South Africa (SA National Accreditation System - ISO 15189:2007).

***Sample identification.*** Blood samples taken will be identified only by the assigned participant identification number given to each participant. This will be attached with a sticker to each blood sample.

***Sample retention at the end of the study.*** Currently no other analyses of stored blood samples are planned. Blood samples will be kept for a period of 3 years after the end of the study and then destroyed. IRB/IEC permission will be obtained before any additional assays are performed on stored samples.

### Quality assurance procedures

***Staff.*** The trial will be conducted in accordance with the current approved protocol, GCP, and trial specific SOPs. All trial staff must hold evidence of appropriate GCP training or undergo GCP training prior to undertaking any responsibilities on this trial. This training should be updated every 2 years. Staff will be trained at the onset of the trial on study requirements; standarised measurement of taking height, weight, and vital signs; counseling for ART initiation and adherence; laboratory specimen collection and processing. All trial requirements and procedures will be explained in detailed SOPs which will be available to staff during the trial. A record of successful trial SOP training will be kept.

***Data.*** All data entered onto source documentation and CRFs will be done in accordance with GCP principles on data quality and integrity. Once the CRF is completed the document needs to be signed and dated. Corrections can only be done by the relevant staff member and must be signed and dated. Source documents and CRFs will be subject to a double quality control check prior to electronic capturing. All electronic capturing of data will also be done in duplicate, with discrepancies resolved in communication between the trial coordinator, relevant data entry personnel, and the clinical staff member responsible for recording the data. Data that is found to be outside a set range of values or is incompatible with a field will be flagged as invalid and discussed with the relevant staff members. Any changes or additions made to source documents or CRFs must be done in accordance with GCP guidelines, recorded on a data-entry and quality control check sheet, and will also be subject to double data entry.

### Ethics

***Ethical conduct.*** This study will be conducted in accordance with the ethical principles laid out in the National Statement on Ethical Conduct in Research Involving Humans, the Declaration of Helsinki (most current version issued) and will be consistent with GCP and applicable regulatory requirements. The study received approval from the University of Cape Town IRB/IEC on 14 January 2019 (HREC Reference: 738/2019). The South African health products regulatory authority (SAHPRA) approved the study on 28 February 2019 (SAHPRA reference number: 20190108). 

The rights, safety, and wellbeing of the study participants are the most important considerations and should prevail over interests of science and society. All personnel involved in conducting this study will be qualified by education, training, and experience to perform their respective tasks.

The IRB/IEC and local regulatory authority approval will be documented and kept in the investigator site file, specifying the version number of the protocol and informed consent as well as the date of approval. Any protocol modifications which may impact on the conduct of the study, affect patient safety, or significant administrative changes will require IRB/IEC and regulatory authority approval. Minor administrative changes which have no effect on trial conduct need only require IRB/IEC and regulatory authority notification. Study documents will be updated in line with the changes implemented in the protocol amendment.

The principal investigator will comply with all IRB/IEC and regulatory authority, reporting requirements for all safety reporting, annual updates, safety updates, end of study reports and any other important information relevant to the conduct of the study.

***Compliance with the protocol.*** The study will be conducted as described in this protocol. The principal investigator will not implement any deviation or change to the protocol without prior review and documented approval/favourable opinion from the IRB/IEC and regulatory authority of an amendment, except where necessary to eliminate an immediate hazard to study participants. Any significant deviation will be reported to the IRB/IEC and regulatory authority.

***Use of placebo.*** The use of a 50 mg dolutegravir (Myltega®) vs placebo in this study will indicate whether the additional 50 mg dose is adequate to achieve and maintain viral suppression. All participants will be on the fixed dose combination antiretroviral tablet of TLD. The phase 2 INSPIRING study ^4^, although proving that the additional dolutegravir dose was well tolerated, was not powered to evaluate the efficacy of the additional dose versus the control arm (efavirenz-based ART) and has therefore not been adopted as standard of care.

***Ancillary and post-trial care.*** Participants will remain on TLD after the trial ends (unless their regimen is changed due to emergent antiretroviral resistance mutations), in line with the South African National Department of Health guidelines for first line ART. This treatment will be supplied by their local community health clinic. If a participant is changed to a different regimen due to emergent antiretroviral resistance mutations, the optimised regimen will also be supplied by their local community health clinic. Any compensation for trial related harm will be paid by the University of Cape Town’s indemnity insurance.

***Confidentiality of data.*** The principal investigator agrees that the University of Cape Town and sponsor, IRB/IEC or regulatory authority may consult and/or copy study documents to verify information in the CRF. By signing the consent form the participant agrees to these processes. 

All study-related information will be stored in secure, locked file cabinets with limited access on the study site. Participant confidentiality will be maintained at all times and no documents containing the participant’s name or other identifying information will be collected. It may be necessary for the sponsor’s representatives, the IRB/IEC and regulatory authority representatives to have direct access to the participant’s medical records. If study documents need to be photocopied during the process of verifying CRF data, the participant will be identified by a unique code only; full names and other identifying information will be masked.

The principal investigator also agrees to maintain confidentiality with all study information and only divulge necessary information to the staff, IRB/IEC, and regulatory authority. The database will be secured with a password protected access system. The data generated by this study will be considered confidential, except where it is included in a publication as agreed in the publication policy of this protocol.

### Monitoring

***Trial committees.*** An IDSMC will be established consisting of three experienced HIV clinical researchers and the study statistician (quorum will be at least two HIV clinical researchers). The members of the IDSMC will be independent of study organisers and serve in an individual capacity to provide expertise and recommendations. The IDSMC will review cumulative study data to evaluate efficacy, safety, study conduct, and scientific validity, and data integrity of the study. The IDSMC will, in strict confidence, review study data biannually. Recommendations are agreed upon by a process of consensus and IDSMC members must be present at the open and closed sessions of the meeting in order to contribute to the letter of recommendation. As this is a non-comparative study, there will be no interim analyses or formal stopping rules – a decision to stop the study for serious harm will be at the discretion of the IDSMC. This IDSMC charter will outline the roles and responsibilities and serve as the SOP for the IDSMC. The findings from the research will be published even if the trial is terminated early based on an IDSMC recommendation.

The TSC will consist of the principal investigator, co-investigators and 2 independent experienced HIV clinicians. The TSC will provide high level oversight and decision-making for the trial (for example, approving the initiation of screening; deciding what action to take following an IDSMC recommendation, etc.) and advise on management of patients who develop virologic failure as per the protocol.

***Independent data monitoring and auditing.*** Independent data monitoring systems with procedures to maximise the quality of every aspect of the study will be implemented. On site data monitoring will be performed by an independent study monitor on a regular basis (scheduling will be a function of patient enrollment and site status). The monitors will:

◦ verify completeness of the investigator site file and regulatory binder
◦ confirm adherence to protocol
◦ review eligibility verification and consent procedures
◦ look for missed clinical event reporting
◦ verify completeness, consistency, and accuracy of data being entered on CRFs
◦ verify completeness, consistency, and accuracy of pharmacokinetic study data and samples
◦ evaluate drug accountability
◦ provide additional training as needed
◦ document findings in a formal feedback letter (monitoring report) to the site

As the trial is registered with SAHPRA, they retain the right to unexpectedly audit the trial and give feedback on any major or minor protocol violations. Should the trial be found unsafe they are within the legal capacity to suspend the trial till such time as the complaints are addressed.

## Dissemination of Information

The trial is registered with Clinicaltrials.gov ( NCT03851588) and SANCTR (DOH-27-072020-8159). The trial protocol will be published in an open-access peer-reviewed journal inaccordance with the Standard Protocol Items: Recommendations for Interventional Trials statement (SPIRIT) ^22^. The trial results will be published in a high-impact open-access journal and presented at relevant international scientific meetings. The trial results will be reported following the Consolidated Standards of Reporting Trials guideline (CONSORT). The results will be disseminated regardless of the magnitude or direction of effect. There will be no restrictions on publication. Authorship will be determined by adequate/equal contribution to the trial conception and design, data collection and analysis, writing, and revision of the manuscript, and final approval of the version for publication. We do not intend to use professional medical writers. Individual patient data will be shared with other originations or individuals for further research upon a reasonable request to the principal investigator, provided that certain conditions are met (including but not limited to the ethical standards upheld by IRB/IEC that approved the original trial).

After the formal publication we will inform relevant health care workers at the recruitment facilities on trial outcomes. Participants that are interested in the trial outcomes or their treatment allocation would be allowed to contact the trial for more information.

## Discussion

Rifampicin induces genes involved in the metabolism and efflux of dolutegravir, resulting in significantly lower dolutegravir exposure. This DDI can be overcome by doubling the dose of dolutegravir to 50 mg 12 hourly, which was well tolerated with good rates of virologic suppression in a phase 2b study of patients with HIV-associated tuberculosis ^4^. However, the additional dose of dolutegravir will be difficult to implement in high burden settings; for this reason, the South African national guidelines on dolutegravir continue to recommend an efavirenz-based ART regimen for ART-naïve or treatment interrupted patients that need to restart first-line ART with tuberculosis.

Four lines of evidence support studying standard dose dolutegravir in patients with HIV-associated tuberculosis. First, the phase 2b study (SPRING-1) of dolutegravir plus dual NRTIs showed similar rates of virologic suppression with dolutegravir dosed at 10 mg, 25 mg, or 50 mg daily and no PK-PD relationships between dolutegravir exposure and virologic outcomes could be established ^6^. Second, the prolonged dissociative half-life of dolutegravir after binding to its receptor ^7^. Third, we conducted a drug-drug interaction study of dolutegravir dosed at 50 mg or 100 mg once daily in healthy volunteers with rifampicin and showed that concomitant rifampicin significantly reduced dolutegravir exposure at both doses, as expected, but all dolutegravir trough concentrations on rifampicin were above the protein-adjusted IC _90_^8^. Furthermore, the geometric mean ratio of the AUC of dolutegravir 50 mg once daily with rifampicin (versus 50 mg daily without rifampicin) was similar to the geometric mean ratio AUC of 10 mg dolutegravir (versus 50 mg dolutegravir), which performed as well as 50 mg daily in the SRING-1 study. Fourth, exposure to the first-generation InSTI raltegravir is also significantly reduced with concomitant rifampicin and, as is the case with dolutegravir, the induction can be overcome by doubling the dose of raltegravir. However, a phase 2 study (ANRS 12 180 Reflate TB) in patients with HIV-associated tuberculosis showed that virologic outcomes were similar with standard and double dose raltegravir ^10^. It is plausible that findings could be similar with dolutegravir.

We propose conducting a phase 2 randomised (1:1) double-blind placebo-controlled trial of the dolutegravir-lamivudine-tenofovir fixed dose combination tablet daily with an additional 50 mg dose of dolutegravir/matching placebo taken 12 hours later in ART-naïve and first-line treatment interrupted PLWH with CD4 counts >100 cells/µL on rifampicin-based antituberculosis therapy. The primary endpoint is virologic suppression at week 24. Key secondary endpoints include the emergence of antiretroviral resistance mutations and dolutegravir trough concentrations. The main risk to participants will be the emergence of antiretroviral resistance mutations, which will be mitigated by frequent viral load monitoring, close monitoring by a data safety monitoring committee, performing genotypic antiretroviral testing in participants with virologic failure, and providing antiretroviral therapy directed by the resistance test results.

Ours will be the first RCT to systematically assess the need for double-dose dolutegravir when treating PLWH on rifampicin-based antituberculosis therapy with a dolutegravir-based ART regimen. The recent programmatic experience from Botswana has shown significant (44%) non-adherence to the recommended double dosing of dolutegravir when prescribed with rifampicin-based antituberculosis therapy ^23^. Even though this approach has revealed very similar virological suppression rates in this population (50 mg dolutegravir once daily: 204/214 (95.3%) versus 50 mg dolutegravir twice daily: 241/254 (94.9%)), the risk of emergent antiretroviral resistance mutations secondary to virological failure should be assessed rigorously before advocating standard dolutegravir dosing among PLWH on rifampicin-based antituberculosis therapy. Our trial would pave the way for a phase 3 randomised control trial to compare standard dolutegravir dosing to an efavirenz-based regimen among PLWH on rifampicin-based antituberculosis therapy.

## Data availability

### Underlying data

No data are associated with this article.

### Extended data

Figshare: RADIANT-TB Informed Consent Form, https://doi.org/10.6084/m9.figshare.13431266.v1 ^11^.

Figshare: RADIANT-TB Mental health assessment questionnaire, https://doi.org/10.6084/m9.figshare.13431305.v1 ^12^.

Figshare: RADIANT-TB Sleep assessment questionnaire, https://doi.org/10.6084/m9.figshare.13431380.v1 ^15^.

### Reporting guidelines

Figshare: SPIRIT checklist for ‘Standard versus double dose dolutegravir in patients with HIV-associated tuberculosis: a phase 2 non-comparative randomised controlled (RADIANT-TB) trial’, https://doi.org/10.6084/m9.figshare.13431137.v1 ^22^.

Data are available under the terms of the Creative Commons Zero "No rights reserved" data waiver (CC0 1.0 Public domain dedication).
