# Supplementary material for: Standard versus double dose dolutegravir in patients with HIV-associated tuberculosis: a phase 2 non-comparative randomised controlled (RADIANT-TB) trial
Source: Wellcome Open Res. 2021 Jan 11;6:1. [Version 1] doi: 10.12688/wellcomeopenres.16473.1 (PMC8063551; doi:10.12688/wellcomeopenres.16473.1)
Supplement: Peer review history

# Peer review: Reviewer response for version 1

**DOI:** 10.21956/wellcomeopenres.18138.r42382

**Published:** 2021-04-14

**Authors:** Kelly Dooley

**Affiliations:** 1 Divisions of Clinical Pharmacology and Infectious Diseases, Department of Medicine, Johns Hopkins University School of Medicine, Baltimore, MD, USA

This is a well-written manuscript describing the design of the RADIANT-TB trial which is being conducted to determine whether or not twice-daily dolutegravir is required when patients are taking rifampicin-based therapy for HIV-associated TB.

 I have only a few comments for the authors' consideration, which are overall minor:

1. The statement that twice-daily treatments are difficult to implement in high burden settings should be supported by references (the example of twice-daily LPV/r, for instance, doesn't have a citation).
2. The rationale for once-daily dosing of DTG with HRZE is strong. The fourth argument the authors make is that raltegravir could be given twice daily but without increasing the dose, and that seemed fine. Were the relationships for trough concentrations of RAL vs. protein-adjusted IC90 similar for RAL in REFLATE and for projected values of DTG in RADIANT?
3. Authors state that positive findings in this trial would lead to organization of a Ph3 trial. But is that needed? Would anyone propose a control arm of EFV in that setting?
4. The inclusion of treatment-naive and also patients who have had interruptions in first-line treatment is a nice feature of this study and adds to its generalizability.
5. When patients have a detectable viral load (at visit 24 and subsequent visits), will they have the opportunity to undergo some adherence counseling and then have a recheck of the viral load?
6. Investigators state that they are measuring tenofovir diphosphate. Is the intent to collect PBMC and measure intracellular TFV-DP? If so, would state that.
7. Investigators state that participants who develop adverse events leading to discontinuation of DTG will be withdrawn from the study. Will they not be followed for final outcomes (including resistance evaluations, where relevant)?
8. Similarly, investigators state that women who become pregnant on-study will be withdrawn and switched to efavirenz-based therapy. Is that still the case, or was that only true earlier on when the study was initially designed? I believe that current standard practice would be to maintain a pregnant woman on dolutegravir (but perhaps at the twice-daily dose), but investigators could comment on that.

Overall, I am enthusiastic about the study. The rationale is strong and the results will be highly relevant for programs. Since the PK-PD for INSTI are still being worked out, this could contribute to our understanding in that realm as well.

Is the study design appropriate for the research question?

Yes

Is the rationale for, and objectives of, the study clearly described?

Yes

Are sufficient details of the methods provided to allow replication by others?

Yes

Are the datasets clearly presented in a useable and accessible format?

Not applicable

Reviewer Expertise:

Pharmacology and therapeutics for TB and HIV.

I confirm that I have read this submission and believe that I have an appropriate level of expertise to confirm that it is of an acceptable scientific standard.

# Peer review: Reviewer response for version 1

**DOI:** 10.21956/wellcomeopenres.18138.r42383

**Published:** 2021-02-24

**Authors:** Derek Sloan

**Affiliations:** 1 Division of Infection and Global Health, School of Medicine, University of St. Andrews, St. Andrews, UK

This is an well-written, important, and timely protocol designed to assess whether standard (once daily) dolutegravir can achieve HIV viral suppression when administered alongside TB therapy in patients who were previously ART-naive or had treatment interrupted (but with low risk of NRTI viral resistance mutations).

 The study is very clearly presented. If it demonstrates that once daily dolutegravir is sufficient to achieve viral suppression during TB therapy, there will be several benefits including: 1) simplicity of medication administration, so that all therapy can be given once per day, and 2) possible reduced side effect / toxicity profile from lower dolutegravir exposure. Collectively, these should make ART adherence easier for PLWH, and reduce the requirement for additional adherence interventions for HIV / TB programmes.

 This is a fairly small study (108 patients), and so is not powered to "compare" single vs. double dose dolutegravir directly. However, a major strength of the proposed work is that the primary end-point is based on a hard, clinically relevant outcome measure (viral load suppression <50 copies/ml). Should this be demonstrated, these data will help to inform policy and guideline change.

 Potential (minor) limitations to the impact of this work include:

1. The possibility that TB treatment regimens will change in the near future to include higher doses of rifampicin, and the effect of this on drug-drug interactions with ART may require ongoing investigation.
2. Recruitment criteria exclude patients who are already established on ART prior to developing TB (though I presume that demonstrating efficacy of single dose dolutegavir will be relevant to they dosing during TB treatment too).
3. The protocol mandates switching patients away from dolutegravir based ART during pregnancy. That is entirely understandable based on South Africa guidelines, which are informed by prior data on the risk of neural tube toxicity. However, in global practice, many women chose to continue dolutegravir based ART throughout pregnancy. WHO, and many international, guidelines now accept use of dolutegravir in pregnancy. Additional work may be needed, in due course, to recommend dosing strategies for HIV-TB management in that setting.
4. There are good adherence monitoring measures built into this study (including use of TFV-DP DBS) which will help to indicate whether differing dolutegravir dosing strategies affect adherence to ART. I'd also be interested to know whether simpler ART makes adherence to concomitant TB therapy easier too (i.e. less pill burden and, maybe, fewer side-effects overall making it easier to take the treatment for both diseases). At present, there are no measurements of this built into the study.

Overall, this is an excellent protocol for a valuable study which will directly inform the management of PLWH who require ART to be co-administered alongside treatment of TB. I'll be excited to see the results emerge.

Is the study design appropriate for the research question?

Yes

Is the rationale for, and objectives of, the study clearly described?

Yes

Are sufficient details of the methods provided to allow replication by others?

Yes

Are the datasets clearly presented in a useable and accessible format?

Not applicable

Reviewer Expertise:

Clinical TB and HIV therapy.

I confirm that I have read this submission and believe that I have an appropriate level of expertise to confirm that it is of an acceptable scientific standard.
